# Exploring medication rules and mechanism of Chinese medicine for children with cough variant asthma based on data mining, network pharmacology, and molecular docking

**DOI:** 10.1097/MD.0000000000040023

**Published:** 2024-10-04

**Authors:** Yuan Ma, Fengping Sun, Yingjie Hu, Jing Li, Yue Ding, Liyang Duan

**Affiliations:** a Department of Traditional Chinese Medicine, Children’s Hospital Affiliated to Zhengzhou University, Henan Children’s Hospital Zhengzhou Children’s Hospital, Zhengzhou, Henan, China.

**Keywords:** Chinese medicine, cough variant asthma in children, data mining, molecular docking, molecular dynamics simulation, network pharmacology

## Abstract

Cough variant asthma (CVA) is a common disease with high incidence among children. Cough is the main clinical symptom and Chinese medicine (CM) has an exact effect on CVA. However, the rules of herb formulation, the pharmacodynamic substances, and the mechanism remained unclear. Therefore, we conducted this article to explore medication rules and molecular mechanism of CM against CVA in children using data mining, network pharmacology, molecular docking, and molecular dynamics simulation. Relevant literatures were collected from China National Knowledge Infrastructure, Chinese Scientific and Technical Journals database, Wanfang database, Pubmed, and Web of science. Excel 2016 was used to extract related data and establish the database for Chinese medical frequency, properties, tastes, and meridian analysis. Association rules were analyzed based on Apriori algorithm using IBM SPSS Modeler 18.0 software and core herb combination was identified. The active ingredients and targets of the core herb combination were acquired form the Traditional Chinese Medicine Systems Pharmacology Database and Analysis Platform database. The main targets of CVA were obtained from the GeneCards and Online Mendelian Inheritance in Man database. Core targets were selected by using STRING platform and Cytoscape 3.7.2 software. Metascape platform was utilized to perform gene ontology and Kyoto Encyclopedia of Genes and Genomes enrichment analysis. The results were verified by molecular docking. SwissADME and pkCSM website were used to analyze the pharmacokinetic profiles and toxicity of key components of the core herb combination. Molecular dynamics simulation was utilized to evaluate the stable of protein–ligand complex. Two hundred seventy-five literatures containing 202 herbs were finally collected. Statistics indicated that these herbs possessed bitter, pungent taste, and warm properties, and belonged to lung meridian. Glycyrrhizae radix et rhizome, Ephedrae herba, and Armeniacae semen amarum were the most frequently used herbs. “Glycyrrhizae radix et rhizoma—ephedrae herba—Armeniacae semen amarum” was the core herb combination with highest support and confidence. Network pharmacology predicted that the main active ingredients, like quercetin, kaempferol, luteolin, etc, might target on RAC-alpha serine/threonine-protein kinase, tumor necrosis factor, interleukin-6, vascular endothelial growth factor A, transcription factor AP-1, interleukin-1 beta, matrix metalloproteinase-9, etc. They played a pivotal role in regulating multiple signaling pathways, such as tumor necrosis factor signaling pathway, IL-17 signaling pathway, and PI3K-Akt signaling pathway. Molecular docking revealed that the key active ingredients were well docked with core targets. The absorption, distribution, metabolism, excretion, and toxicity analysis showed that formononetin, luteolin, naringenin, and quercetin have high gastrointestinal absorption, no AMES toxicity, hepatotoxicity, and skin sensitization. Molecular dynamics simulation revealed that the formononetin–matrix metalloproteinase-9 complex was relatively stable. This article revealed that CM against CVA in children focused on dispelling wind and reducing phlegm, warming lung, and relieving cough. The mechanism of the core herb combination of CM for CVA through muti-components, muti-targets, and muti-pathways.

## 1. Introduction

Cough variant asthma (CVA) is a form of atypical asthma characterized by chronic cough without dyspnea or wheezing.^[1,2]^ CVA often occurs at night or early in the morning. It is the main cause of chronic cough in children and seriously affects the physical and mental health of children. According to epidemiological data, the incidence rate of CVA in children has been going up in recent years.^[3]^ Inhaled corticosteroids, bronchodilators, and leukotriene receptor antagonists are the primary treatments for children with CVA. However, there are numerous adverse effects, lengthy treatment courses, and a high risk of disease recurrence following medication withdrawal. In addition, persistent CVA may progress to conventional asthma once treatment is discontinued.^[4,5]^

**Figure 1.**
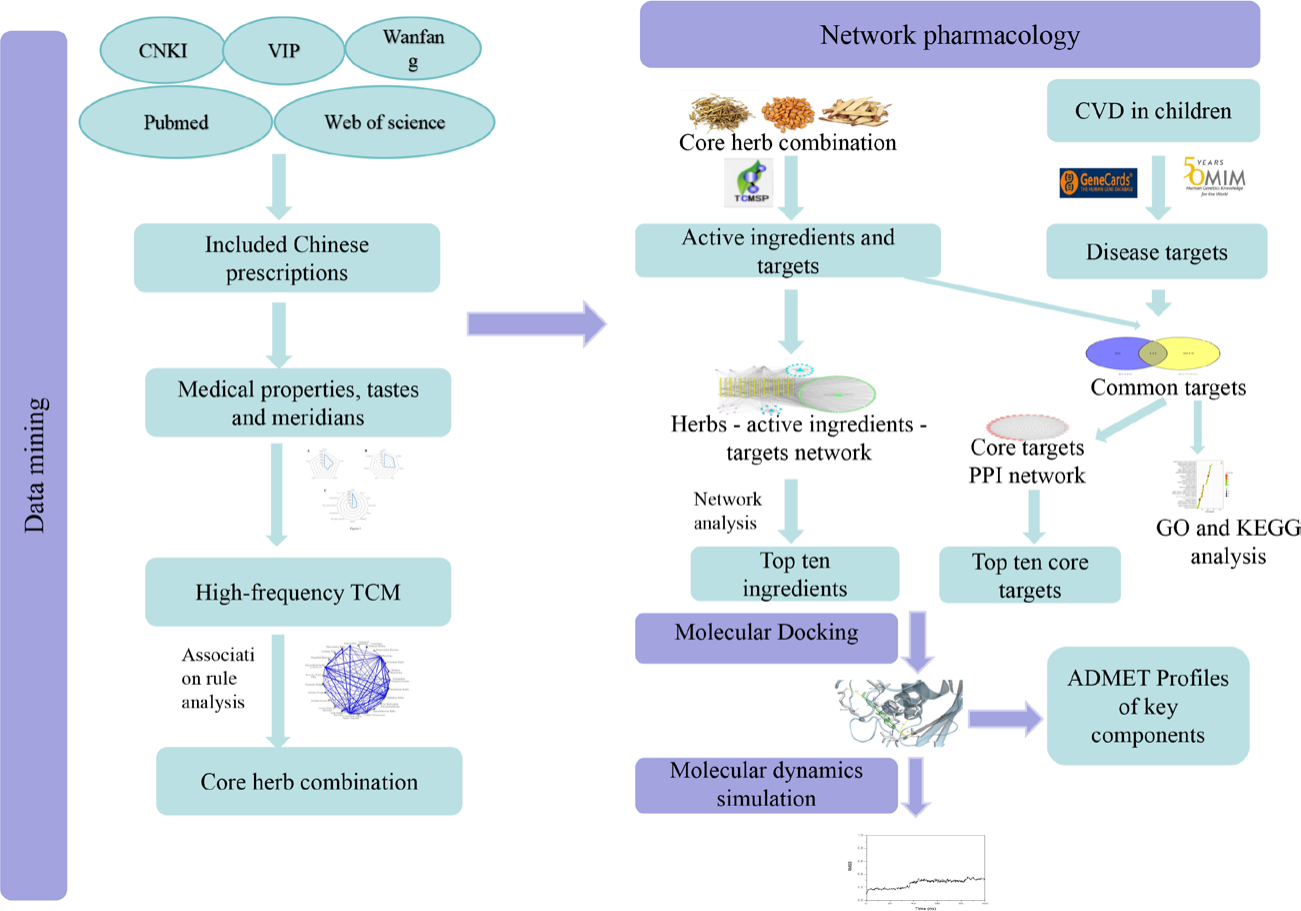
The framework of this article.

Chinese medicine (CM) has a distinct advantage in the treatment of children with CVA because of remarkable curative effect and less side-effects.^[6,7]^ However, the prescriptions of CM are mostly derived from personal clinical experience. The compatibility principle of CM, the basis of pharmacodynamic substances and the mechanism have not been clarified. Based on the above consideration, this article was aimed to explore the prescription medication rules of CM against CVA in children by extensively collecting and analyzing the relevant literatures. We also explored the principle of core herb combination using data mining. Network pharmacology, molecular docking, and molecular dynamics simulation were used for exploring mechanism of the core herb combination. This article provided theoretical basis for the subsequent clinical and experimental studies. The framework of this article was presented in Figure 1.

## 2. Materials and methods

### 2.1. Data mining

#### 2.1.1. Data source

China National Knowledge Infrastructure, Chinese Scientific and Technical Journals Database (VIP Database), Wan-fang Database, PubMed, and Web of Science were used to retrieve literatures involving TCM treatment of children with CVA for the time period from the creation of each database until March 10, 2023. We conducted a systematic search using the following search strategy: (“cough variant asthma” OR “cough type asthma” OR “CVA”) AND (“children” OR “adolescent” OR “infant” OR “young” or “preschool”) AND (“Chinese medicine” OR “Chinese herb” OR “Traditional Chinese medicine” OR “TCM”).

#### 2.1.2. Inclusion criteria

① The type of study must be a prospective clinical study (randomized controlled trial or not); ② all of the selected literatures met the CVA diagnostic criteria in the Guidelines for Diagnosis and Prevention of Bronchial Asthma in Children (2016 Edition).^[8]^ ③ The participants were children with CVA < 18 years old; ④ clear prescription; ⑤ curative effect was exact and the total clinical effective rate was ≥80%.

#### 2.1.3. Exclusion criteria

① Duplicate and single herb literatures; ② Chinese patent medicine, paste prescription and other special prescriptions (non-decoction oral dosage forms); ③ combined with other nondrug treatments (acupoint application, acupuncture, cupping, and so on); ④ studies in cells and animals; ⑤ review and theoretical research literatures; ⑥ among the publications using the same data, only the most relevant literature would be remained.

#### 2.1.4. Literature selection and data extraction

The selected literatures were imported into Endnote X9 to remove duplication. Two reviewers independently selected related literatures after reading the titles, abstracts, and keywords according to above accepting criteria. The remaining documents were screened after reading the full text. To establish the database of CM treatment for children with CVA, 2 researchers extracted data independently utilizing Excel 2016, including the article title, researchers’ name, prescription name, drug composition, etc.

#### 2.1.5. Standardization of TCM names

According to the Pharmacopoeia of the People’s Republic of China (2020 edition) and Chinese Materia Medica, the names of TCM were standardized.

#### 2.1.6. Data statistics and analysis

Excel 2016 was utilized to conduct statistics on the frequency of properties, tastes and meridians of CM included in prescription database, and generate radar charts of properties, tastes and meridians. To investigate the core drug compatibility of CM for CVA in children, an association rule analysis of herbs with high-frequency (frequency > 40) was performed using Apriori algorithm in IBM SPSS Modeler 18.0 software. The parameters was used to set up the minimum rule confidence of 80%, the minimum support of 10%, and the maximum number of antecedents of 2.

### 2.2. Network pharmacology

#### 2.2.1. Active ingredients and targets of core herb combination screening

The active ingredients of the core herb combination were found through the Traditional Chinese Medicine Systems Pharmacology Database and Analysis Platform (https://old.tcmsp-e.com/tcmsp.php). The active ingredients in compliance with the requirements of oral bioavailability ≥ 30% and drug-likeness ≥ 0.18 were screened. After standardizing the targets name by using the UniProt website (https://www.uniprot.org/). The herbs-active components-targets network diagram was set up and the key active ingredients were screened by network analysis.

#### 2.2.2. Screening the targets of CVA in children

The key word “CVA in children” was utilized to search in the GeneCards database (https://www.genecards.org/) and Online Mendelian Inheritance in Man database (https://www.omim.org/) for disease targets after merging and deleting duplication.

#### 2.2.3. Core targets screening and protein–protein interaction (PPI) network construction

The Venny 2.1.0 online software (https://bioinfogp.cnb.csic.es/tools/venny/index.html) was utilized to screen common targets between herbs and disease, and then common targets were imported into the STRING 11.5 website (https://www.string-db.org/) for PPI network construction. Then the network data was loaded into the Cytoscape 3.7.2 software for topological analysis. The core targets were those that satisfied the medians of degree, betweenness centrality, and closeness centrality of topological eigenvalue nodes and Cytoscape 3.7.2 was used to visualize the results.

#### 2.2.4. Gene ontology (GO) and Kyoto Encyclopedia of Genes and Genomes (KEGG) enrichment analyses

Metascape platform (https://metascape.org/gp/index.html) was utilized to perform GO and KEGG enrichment analysis. Network visualization was used by bioinformatics online software (https://www.bioinformatics.com.cn/).

### 2.3. Molecular docking

The key active ingredients (top 10 degrees in the herbs-active components-targets network) and core targets (top 10 degrees in the PPI network) were used for molecular docking. Two-dimensional structures of key active ingredients structure were from Pubchem database (https://pubchem.ncbi.nlm.nih.gov/). Ligands were saved in mol2 format using Open Babel 2.4.1 software. Download the core targets protein structure from RSCBPDB database (https://www.rcsb.org/). The screening conditions were set as follows: ① the protein with low resolution and screened with the preferred X-ray method was preferred; ② the species derived from Homo sapiens; ③ the protein with original ligands was selected. The original ligands and water molecules of proteins were removed using PyMoL2.3.2. Then hydrogen atoms and charge operations of proteins were added by AutoDock Tools. AutoDock vina was applied for docking. The original ligand binding site was used as the protein binding site, and if in the absence of the original ligand, the reported key amino acid residues were set as the docking area. Meanwhile, the docking box was adjusted to include all protein structures. The method of semi flexible docking was applied. Protein–ligand interaction profiler (https://plip-tool.biotec.tu-dresden.de) and PyMoL2.3.2 were utilized to analyze and visualize the interactions between the key active ingredients and core targets.

### 2.4. The pharmacokinetic profile and toxicity of key components of the core herb combination

The pharmacokinetic parameters of the absorption, distribution, metabolism, excretion, and toxicity (ADMET) analysis was an important tool in drug discovery. Pharmacokinetic profiles and toxicity profile of key components of the core herb combination were predicted by using SwissADME website (http://www.swissadme.ch/) and pkCSM website (https://biosig.lab.uq.edu.au/pkcsm/).

### 2.5. Molecular dynamics simulation

Gromacs2021 was selected as the dynamics simulation (molecular dynamics, MD), and Amber was selected as the protein and small molecule force field. Use the SPCE model to add water to the system, establish a water box of 10 × 10 × 10 nm^3^ (the edge of the water box was at least 1.2 nm away from the edge of the protein), and add an ion automatic balance system. Particle-mesh Ewald (PME) handles electrostatic interactions and uses the steepest descent method for energy minimization for a maximum number of steps (50,000 steps). The Coulomb force cutoff distance and the van der Waals radius cutoff distance were both 1 nm. Finally, the NVT and the NPT were used to balance the system, and then the 100 ns MD simulation was performed at normal temperature and pressure. The nonbonded interaction cutoff was set to 10 Å. The V-rescale temperature coupling method was used to control the simulation temperature to 300 K, and the Berendsen method was used to control the pressure to 1 bar. The built-in analysis module of Gromacs2021 was used to conduct data analysis. Root mean square deviation (RMSD) was used to observe the overall changes of the protein relative to the initial structure of the system during the simulation process. Radius of gyration (Rg) was used to evaluate the tightness of the system. Root mean square function (RMSF) was utilized to observe the structure fluctuations of local amino acid residue sites in the system during the simulation process. The binding free energy of protein-ligand was analyzed and calculated by the MM/PBSA.

## 3. Results

### 3.1. Data mining

#### 3.1.1. Data screening

**Figure 2.**
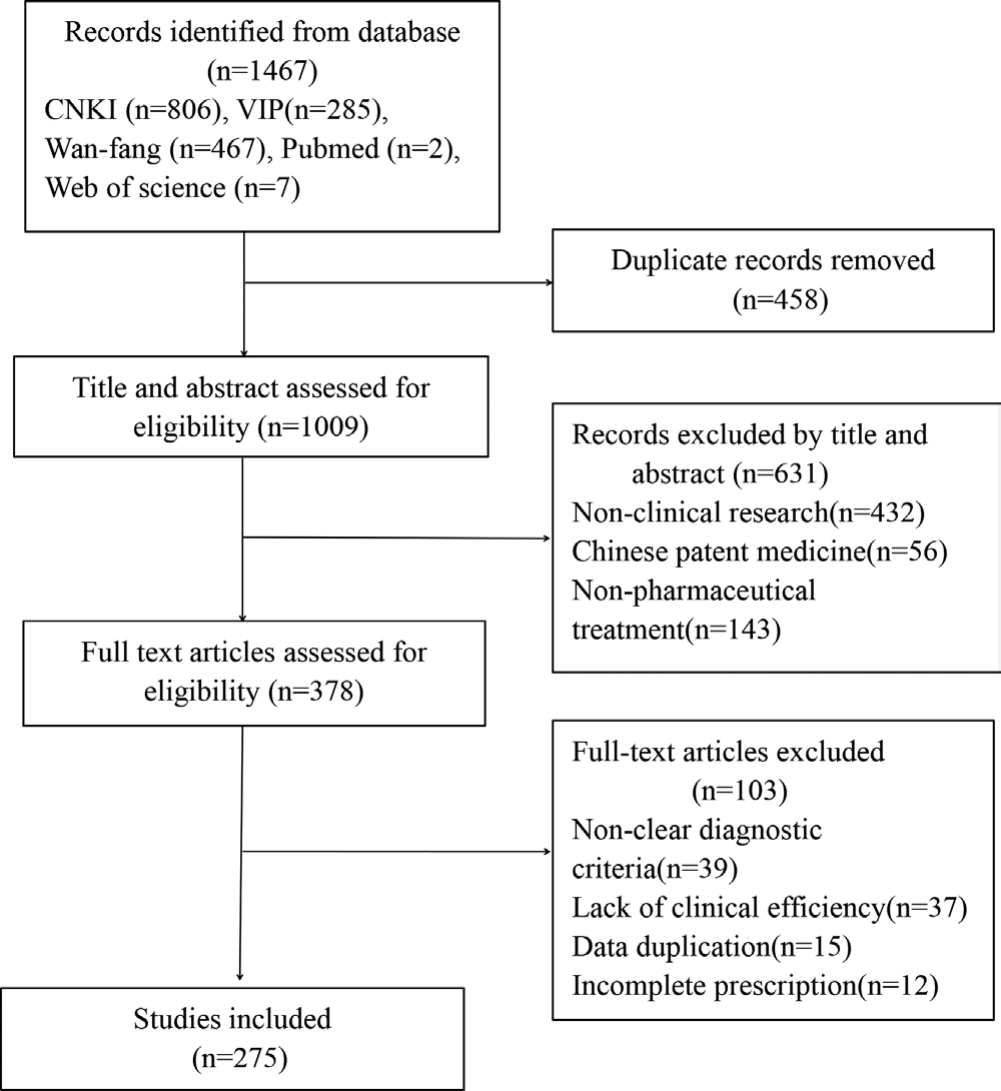
Flow chart of the literature selection process.

In total, 1467 literatures were obtained and 1009 articles remained after removing duplicate studies by Endnote X9.3. Following an evaluation of the titles and abstracts, 378 studies were included. Ultimately, 275 studies were collected after reading the full text. The detailed selection process was illustrated in Figure 2.

#### 3.1.2. Frequency of properties, tastes and meridians of CM

**Figure 3.**
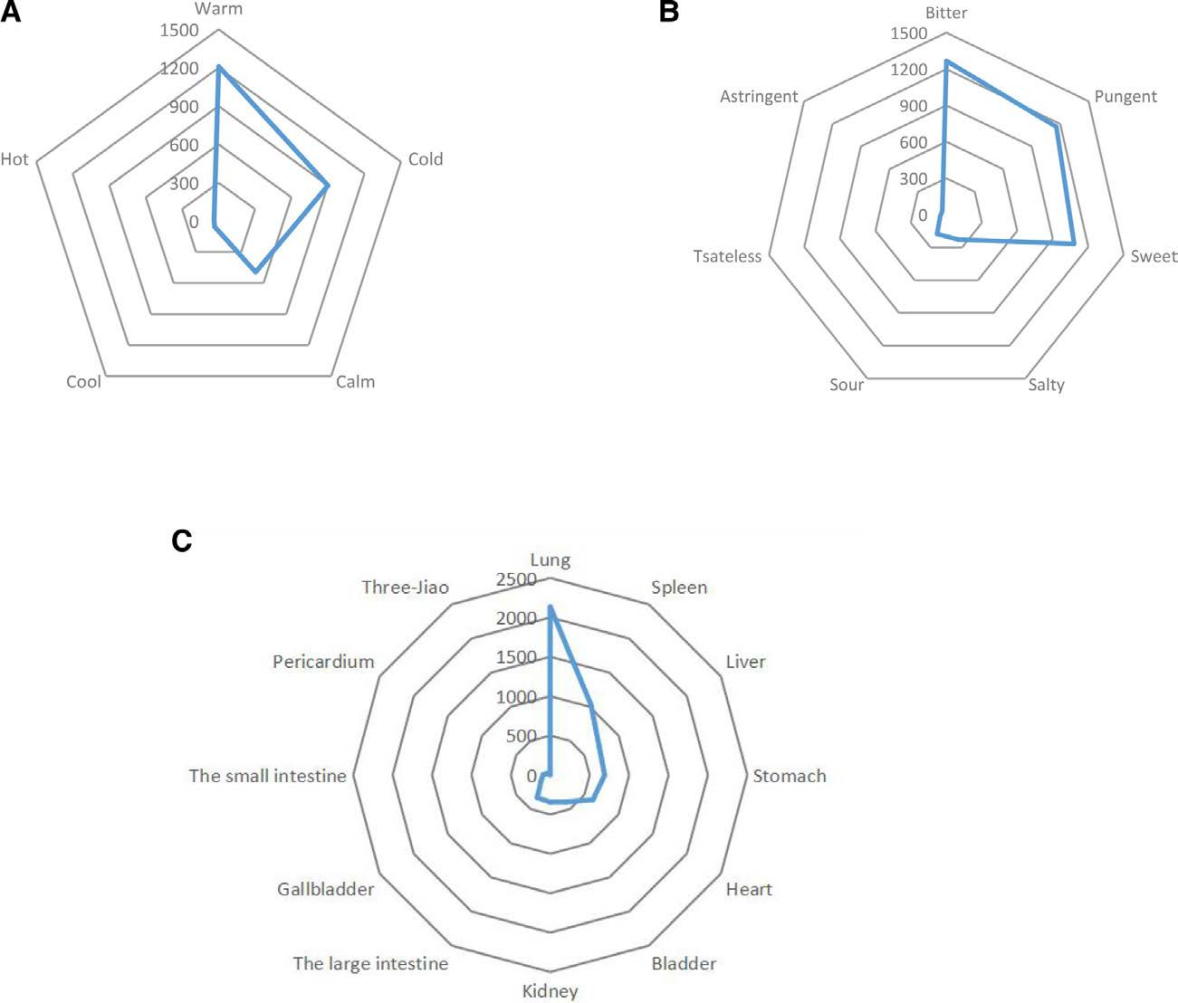
Frequency of properties, tastes and meridians of CM in 275 prescriptions. (A) Properties of CM; (B) tastes of CM; (C) meridians of CM. CM = Chinese medicine.

After data collection and analysis, a total of 275 prescriptions containing 202 herbs were finally collected and the cumulative frequency of herbs amounted to 2694 times. Ephedrae herba (Mahuang) was utilized 155 times (5.75%) among the 20 most frequently used CM followed by armeniacae semen amarum (Kuxingren)152 times (5.64%) and licorice (Gancao) 129 times (4.79%), as shown in Table 1. The analysis of the properties, tastes and meridians of CM revealed that the properties were mainly warm (1210 times, 44.85%), cold (901 times, 33.4%), and calm (494 times, 18.31%), the tastes were mostly bitter (1268 times, 31.61%), pungent (1161 times, 28.94%), and sweet (1081 times, 26.94%), and the main meridians are lung (2133 times, 32.62%), spleen (1031 times, 15.77%), liver (717 times, 10.96%), and stomach (689 times, 10.54%), as shown in Figure 3.

**Table 1:** The high-frequency of CM in prescriptions (frequency ≥ 40 times).

Chinese name	Latin name	Properties and tastes	Meridian	Frequency	Percentage (%)
Mahuang	Ephedrae Herba	Warm; pungent, bitter	Lung, bladder	155	5.75
Kuxingren	Armeniacae Semen Amarum	Warm; bitter	Lung, the large intestine	152	5.64
Gancao	Glycyrrhizae Radix Et Rhizoma	Calm; sweet	Heart, lung, spleen, stomach	129	4.79
Dilong	Pheretima	Cold; salty	Liver, spleen, bladder	123	4.57
Chantui	Cicadae Periostracum	Cold; sweet	Lung, liver	97	3.6
Wuweizi	Schisandrae Chinensis Fructus	Warm; sour, sweet	Lung, heart, kidney	77	2.86
Huangqin	Scutellariae Radix	Cold; bitter	Lung, gallbladder, spleen, the large intestine, the small intestine	74	2.75
Jiegeng	Platycodonis Radix	Calm; bitter, pungent	Lung	67	2.49
Ziwan	Asteris Radix Et Rhizoma	Warm; pungent, bitter	Lung	61	2.26
Baibu	Stemonae Radix	Warm; sweet, bitter	Lung	58	2.15
Sangbaipi	Mori Cortex	Cold; sweet	Lung	57	2.12
Xixin	Asari Radix Et Rhizoma	Warm; pungent	Heart, lung, kidney	56	2.08
Chenpi	Citri Reticulatae Pericarpium	Warm; bitter,pungent	Lung, spleen	55	2.04
Baishao	Paeoniae Radix Alba	Cold; bitter, sour	Liver, spleen	50	1.86
Fangfeng	Saposhnikoviae Radix	Warm; pungent, sweet	Bladder, liver, spleen	49	1.82
Fuling	Poria	Calm; sweet, tasteless	Heart, lung, spleen, kidney	46	1.71
Jiangcan	Bombyx Batryticatus	Calm; salty, pungent	Liver, lung, stomach	46	1.71
Qianhu	Peucedani Radix	Cold; bitter, pungent	Lung	45	1.67
Zisuye	Perillae Folium	Warm; pungent	Lung, spleen	44	1.63
Kuandonghua	Farfarae Flos	Warm; pungent, bitter	Lung	42	1.56

CM = Chinese medicine.

#### 3.1.3. Association rule analysis

**Figure 4.**
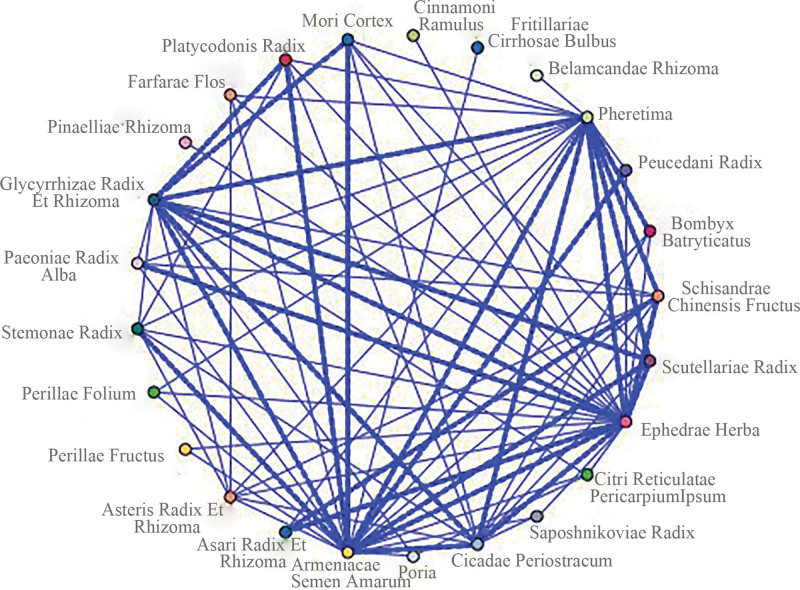
Network diagram of herbal combination patterns.

Combined with the ranking of frequency, support and confidence, core herb combinations were Glycyrrhizae Radix Et Rhizoma (Gancao)–Ephedrae Herba (Mahuang)–Armeniacae Semen Amarum (Kuxingren), Asari Radix Et Rhizoma (Xixin)–Ephedrae Herba (Mahuang), Scutellariae Radix (Huangqin)–Armeniacae Semen Amarum (Kuxingren)–Ephedrae Herba (Mahuang), as shown in Table 2. Glycyrrhizae Radix Et Rhizoma (Gancao)–Ephedrae Herba (Mahuang)–Armeniacae Semen Amarum (Kuxingren) with the highest support and confidence among them, was chosen as the core herb combination for follow-up research. The association network among high-frequency CM was shown in Figure 4.

**Table 2:** Herbal combinations for treatment of CVA in children.

Herbal combination	Frequency	Support (%)	Confidence (%)
Glycyrrhizae Radix Et Rhizoma(Gancao)–Ephedrae Herba(Mahuang)–Armeniacae Semen Amarum (Kuxingren)	84	32.68	90.38
Asari Radix Et Rhizoma (Xixin)–Ephedrae Herba (Mahuang)	56	21.79	82.14
Scutellariae Radix (Huangqin)- Armeniacae Semen Amarum (Kuxingren) -Ephedrae Herba (Mahuang)	54	21.01	87.04

CVA = cough variant asthma.

### 3.2. Results of network pharmacology

#### 3.2.1. Active ingredients and targets of core herb combination

**Figure 5.**
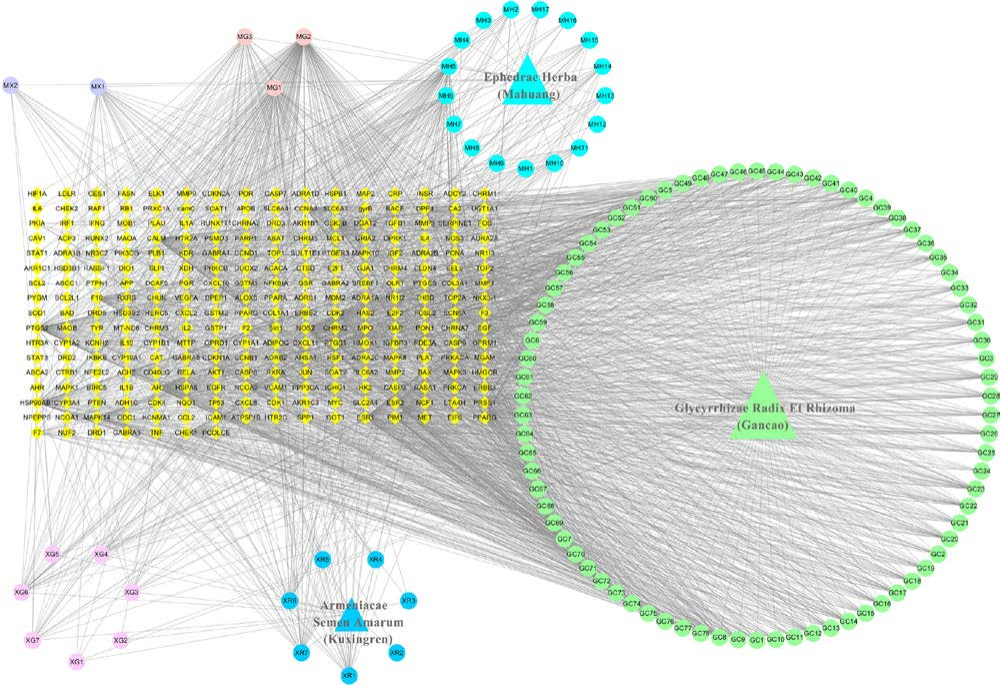
Network diagram of herbs—active ingredients—targets of core herb combination against CVA in children. CVA = cough variant asthma.

One hundred fourteen active ingredients meeting the requirements were screened, and their related targets were further explored and standardized through Uniprot database. Two hundred sixty-three targets were contained after weight removal. Calculated the degree after importing Cytoscape 3.7.2 software, and the first 15 active ingredients with degree value > 30 were screened out, as shown in Table 3. The network diagram of herbs—active ingredients—targets was constructed and this network contained 380 nodes and 2225 edges, as shown in Figure 5.

**Table 3:** Key active ingredients of core herb combination in treating CVA in children (degree > 30).

No.	Mol ID	Compound name	Degree	OB (%)	DL	Source of the herb
1	MOL000098	Quercetin	154	46.43	0.28	Ephedrae Herba (Mahuang), Glycyrrhizae Radix Et Rhizoma (Gancao)
2	MOL000422	Kaempferol	63	41.88	0.24	Ephedrae Herba (Mahuang), Glycyrrhizae Radix Et Rhizoma (Gancao)
3	MOL000006	Luteolin	57	36.16	0.25	Ephedrae Herba (Mahuang)
4	MOL003896	7-Methoxy-2-methyl isoflavone	43	42.56	0.2	Glycyrrhizae Radix Et Rhizoma (Gancao)
5	MOL000392	Formononetin	39	69.67	0.21	Glycyrrhizae Radix Et Rhizoma (Gancao)
6	MOL000358	Beta-sitosterol	38	36.91	0.75	Ephedrae Herba (Mahuang)
7	MOL000354	Isorhamnetin	37	49.6	0.31	Glycyrrhizae Radix Et Rhizoma (Gancao)
8	MOL004328	Naringenin	37	59.29	0.21	Ephedrae Herba (Mahuang), Glycyrrhizae Radix Et Rhizoma (Gancao)
9	MOL002565	Medicarpin	34	49.22	0.34	Glycyrrhizae Radix Et Rhizoma (Gancao)
10	MOL000497	Licochalcone a	32	40.79	0.29	Glycyrrhizae Radix Et Rhizoma (Gancao)
11	MOL004978	2-[(3R)-8,8-dimethyl-3,4-dihydro-2H-pyrano[6,5-f]chromen-3-yl]-5-methoxyphenol	31	36.21	0.52	Glycyrrhizae Radix Et Rhizoma (Gancao)
12	MOL000449	Stigmasterol	31	43.83	0.76	Ephedrae Herba (Mahuang), Armeniacae Semen Amarum (Kuxingren)
13	MOL012922	l-SPD	30	87.35	0.54	Armeniacae Semen Amarum (Kuxingren)
14	MOL004891	Shinpterocarpin	30	80.3	0.73	Glycyrrhizae Radix Et Rhizoma (Gancao)
15	MOL000500	Vestitol	30	74.66	0.21	Glycyrrhizae Radix Et Rhizoma (Gancao)

CVA = cough variant asthma, DL = drug-likeness, OB = oral bioavailability.

#### 3.2.2. Targets of CVA in children

**Figure 6.**
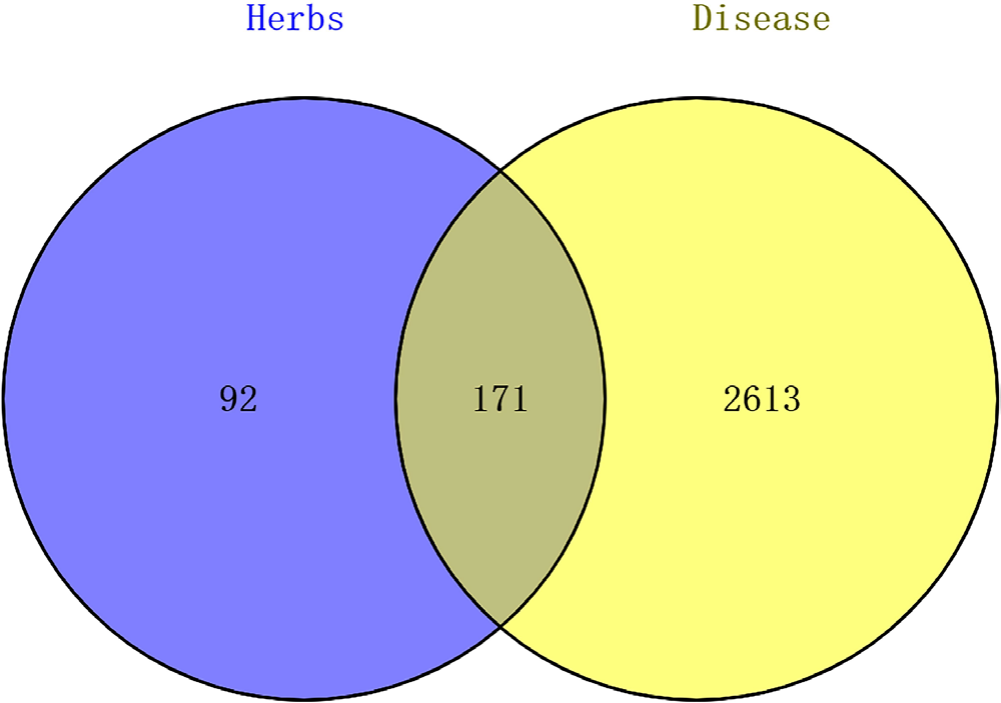
Venn diagram of common targets of herbs and disease.

Ultimately, a total of 2824 targets were obtained from Genecards and Online Mendelian Inheritance in Man databases after removing duplicates. One hundred seventy-one common targets were identified by intersecting herb targets and disease targets using Venny2.1.0, as shown in Figure 6.

#### 3.2.3. PPI network construction and core targets selection

**Figure 7.**
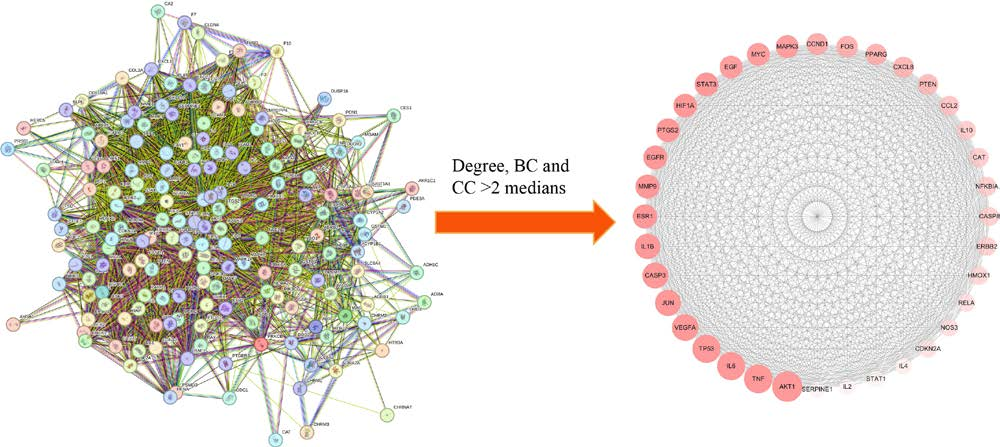
PPI network and hub targets. PPI = protein–protein interaction.

One hundred seventy-one common targets were imported into STRING 11.5 database to build PPI network and this network involved 171 nodes and 3720 edges. CytoNCA was utilized to identify important targets and 36 core targets were screened out, as shown in Figure 7. Among the core targets, the top 10 targets were RAC-alpha serine/threonine-protein kinase (AKT1), tumor necrosis factor (TNF), interleukin-6 (IL6), tumor protein P53, vascular endothelial growth factor A (VEGFA), transcription factor AP-1 (JUN), caspase 3, interleukin-1 beta (IL1B), estrogen receptor 1, and matrix metalloproteinase-9 (MMP9).

#### 3.2.4. GO and KEGG enrichment analysis

**Figure 8.**
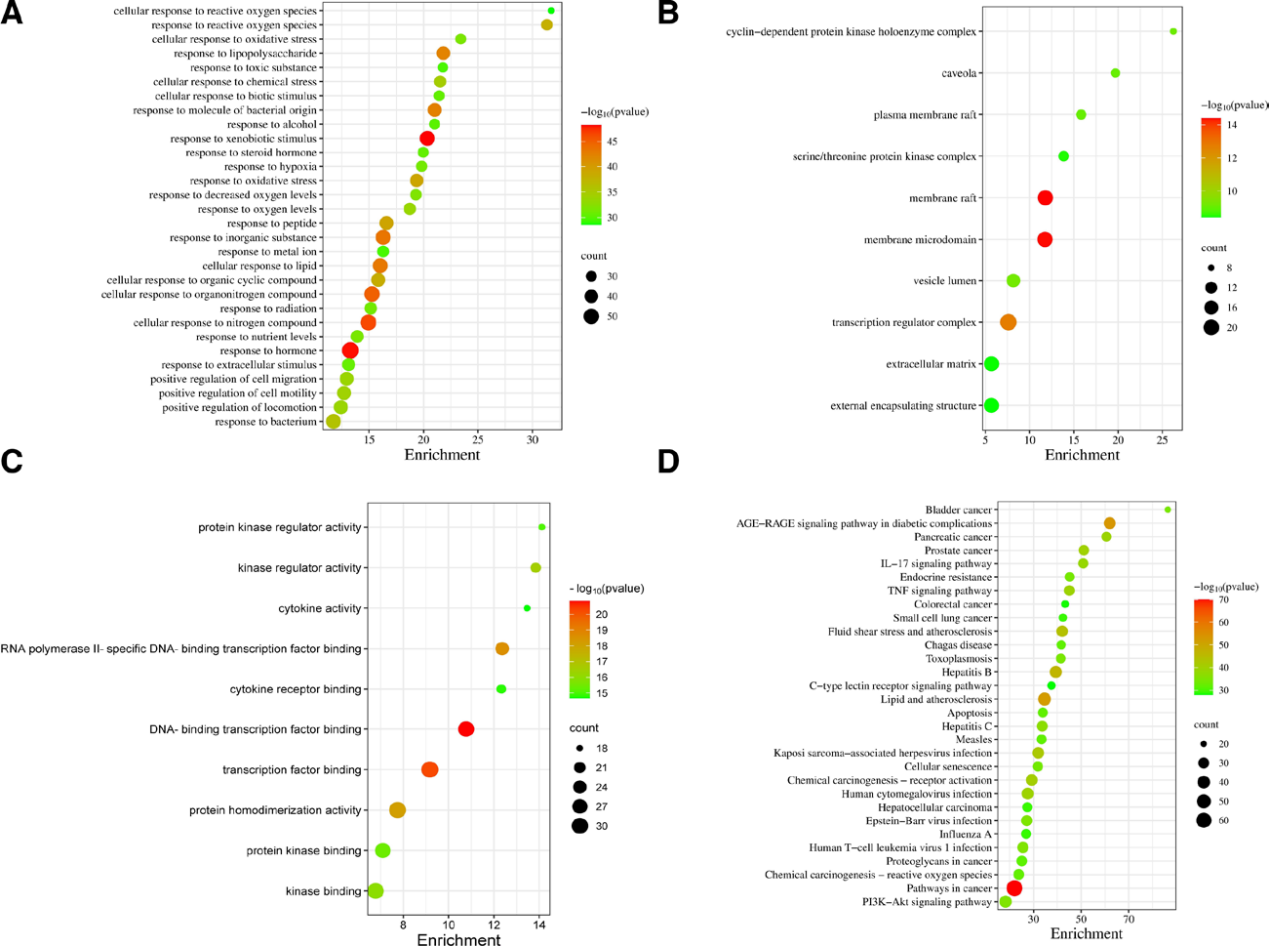
Gene enrichment analysis. (A) Go biological process; (B) Go cellular components; (C) Go molecular function; (D) KEGG analysis. GO = gene ontology, KEGG = Kyoto Encyclopedia of Genes and Genomes.

A total of 2221 GO enrichment items were displayed. Among them, 1945 items in biological process (BP) were identified, such as response to lipopolysaccharide, response to oxidative stress, response to hypoxia and response to metal ion. Cellular component was made up of 91 items, including cell membrane, transcription regulator complex, extracellular matrix, and so on. Molecular function was consisted of 155 items, including transcription factor binding, protein kinase regulator activity, cytokine activity, etc. The first 30 items in BP and 10 terms in closeness centrality, molecular function were selected for visualization, as shown in Figure 8A–C.

Two hundred three signal pathways were enriched after KEGG analysis and the main items included TNF signaling pathway, IL-17 signaling pathway, PI3K-Akt signaling pathway, apoptosis, human cytomegalovirus infection, and Epstein-Barr virus infection. As depicted in Figure 8D, the first 30 items were selected for visualization.

### 3.3. Molecular docking and ADMET profiling

**Figure 9.**
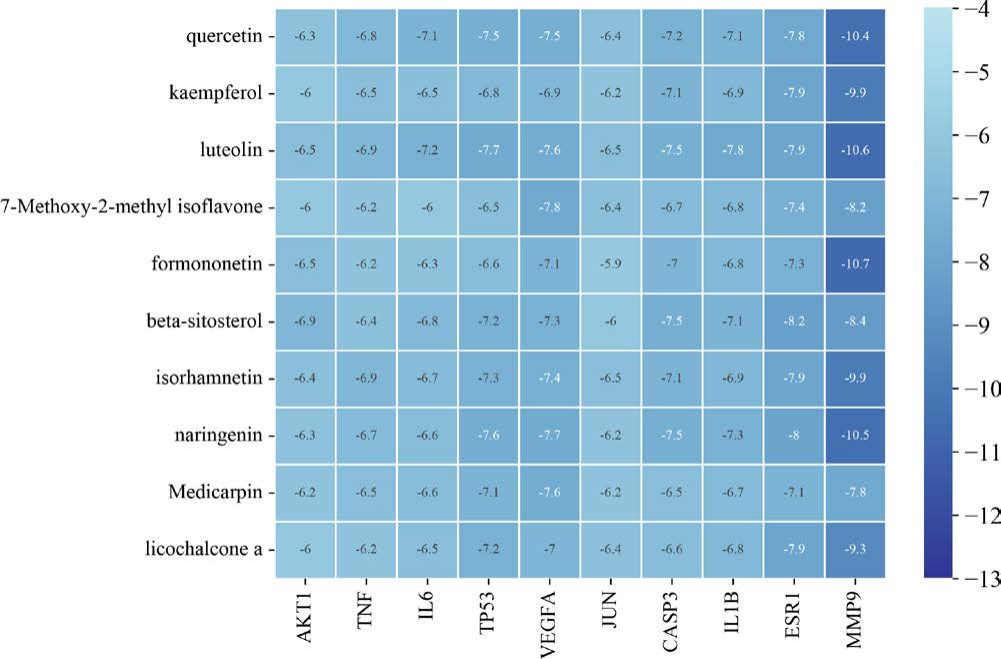
Molecular docking results of active ingredients and key targets.

**Figure 10.**
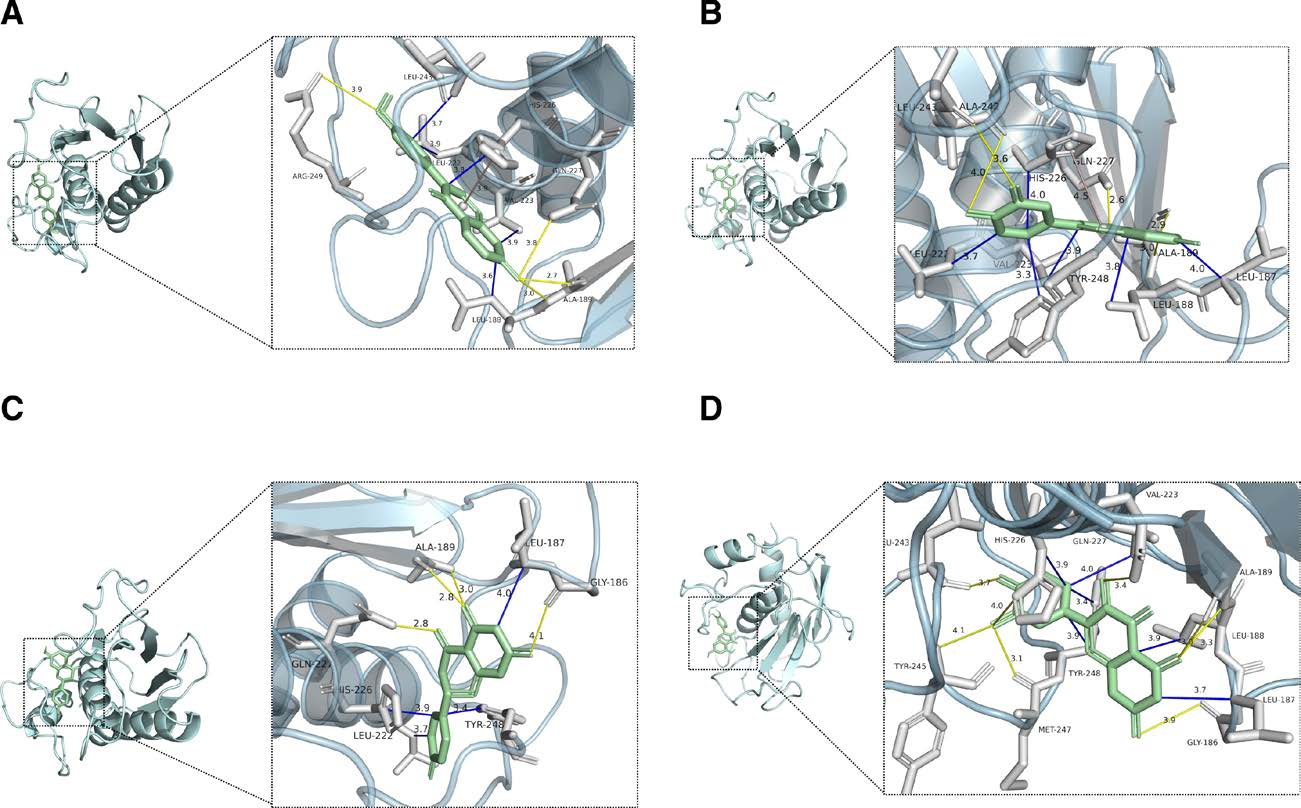
Molecular docking modes of formononetin, luteolin, naringenin, quercetin and MMP9. (A) formononetin–MMP9, (B) luteolin–MMP9, (C) naringenin–MMP9, (D) quercetin–MMP9. MMP9 = matrix metalloproteinase-9.

Autodock vina was used for molecular docking between the top 10 key active ingredients (quercetin, kaempferol, luteolin, 7-methoxy-2-methyl isoflavone, formononetin, beta-sitosterol, isorhamnetin, naringenin, medicarpin, licochalcone a) and the top 10 core targets (AKT1 [PDBID:1UNQ], TNF [PDBID:5UUI], IL6 [PDBID:1ALU], tumor protein P53 [PDBID:3D06], VEGFA [PDBID:1MKK], JUN [PDBID:6Y3V], caspase 3 [PDBID:2DKO], IL1B [PDBID:5R8Q], estrogen receptor 1 [PDBID:7BAA], and MMP9 [PDBID:6ESM]). It is widely assumed that the lower the binding energy, the more stable the ligand-receptor binding configuration, therefore the binding energy < ‐4.25 kcal/mol can be deemed to have binding activity. Binding energy < ‐5.0 kcal/mol indicates good binding activity, while binding energy <‐7.0 kcal/mol denotes strong binding activity.^[9,10]^ All of the docking scores were <‐5 kcal/mol, indicating that these 10 active components could stably bind with core target proteins, as shown in Figure 9. The docking scores of formononetin, luteolin, naringenin, and quercetin with MMP9 were all <‐10 kcal/mol, which suggested that formononetin, luteolin, naringenin, and quercetin might be key components of the core drug combination, and MMP9 was an important target for intervention CVA in children. The detailed binding energies of formononetin, luteolin, naringenin, and quercetin with MMP9 was shown in Figure 10 and Table 4.

**Table 4:** Binding energies of the key active components to MMP9.

Component-target	Docking score (kcal/mol)	Interaction					
		Hydrogen bond		Hydrophobic interaction		π-cation interaction	
		Amino acid	Distance (Å)	Amino acid	Distance (Å)	Amino acid	Distance (Å)
Formononetin-MMP9	‐10.7	ALA-189	3.0	LEU-188	3.6	HIS-226	3.9
		ALA-189	2.7	LEU-222	3.9		
		GLN-227	3.8	VAL-223	3.9		
		ARG-249	3.9	HIS-226	3.8		
				LEU-243	3.7		
Luteolin-MMP9 complex	‐10.6	ALA-189	3.0	LEU-187	4.0	HIS-226	4.9
		ALA-189	2.9	LEU-188	3.8		
		GLN-227	2.6	LEU-222	3.7		
		ALA-242	3.6	VAL-223	3.9		
		LEU-243	4.0	HIS-226	4.0		
				TYR-248	3.3		
Naringenin-MMP9	‐10.5	GLY-186	4.1	LEU-187	4.0		
		ALA-189	3.0	LEU-222	3.7		
		ALA-189	2.8	HIS-226	3.9		
		GLN-227	2.8	TYR-248	3.4		
Quercetin-MMP9 complex	‐10.4	GLY-186	3.9	LEU-187	3.7		
		ALA-189	3.3	LEU-188	3.9		
		ALA-189	3.0	VAL-223	4.0		
		HIS-226	4.0	HIS-226	3.9		
		GLN-227	3.4	TYR-248	3.9		
		LEU-243	3.7	TYR-248	3.4		
		TYR-245	4.1				
		MET-247	3.1				

CVA = cough variant asthma, MMP9 = matrix metalloproteinase-9.

As shown in Table 5, the ADME analysis utilizing the SwissADME exhibited formononetin, luteolin, naringenin, and quercetin showed high gastrointestinal absorption. The blood-brain barrier permeant was found in formononetin and Pap substrate was found in naringenin. Formononetin, luteolin, naringenin, and quercetin inhibited isoforms CYP1A2 and CYP3A4 ability. Formononetin, luteolin, and quercetin inhibited isoforms CYP2D6 ability. As shown in Table 6, the toxicity of key active ingredients utilizing the pkCSM website showed that formononetin, luteolin, naringenin, and quercetin had no AMES toxicity, hepatotoxicity, and skin sensitization. All of them were inhibitors of HERG I and HERG II.

**Table 5:** Pharmacokinetic properties of key active ingredients.

Compound name	GI absorption	BBB permeant	Pap substrate	CYP1A2 inhibitor	CYP2C19 inhibitor	CYP2C9 inhibitor	CYP2D6 inhibitor	CYP3A4 inhibitor
Formononetin	High	Yes	No	Yes	No	No	Yes	Yes
Luteolin	High	No	No	Yes	No	No	Yes	Yes
Naringenin	High	No	Yes	Yes	No	No	No	Yes
Quercetin	High	No	No	Yes	No	No	Yes	Yes

BBB = blood-brain barrier, GI = gastrointestinal.

**Table 6:** Predicted toxicity of key active components.

Compound name	AMES toxicity	HERG I inhibitor	HERG II inhibitor	Hepatotoxicity	Skin sensitisation	Max tolerated dose (log mg/kg/day)	Minnow toxicity (log mM)
Formononetin	No	No	No	No	No	0.008	0.041
Luteolin	No	No	No	No	No	0.499	3.169
Naringenin	No	No	No	No	No	‐0.176	2.136
Quercetin	No	No	No	No	No	0.499	3.721

### 3.4. Molecular dynamics simulation

**Figure 11.**
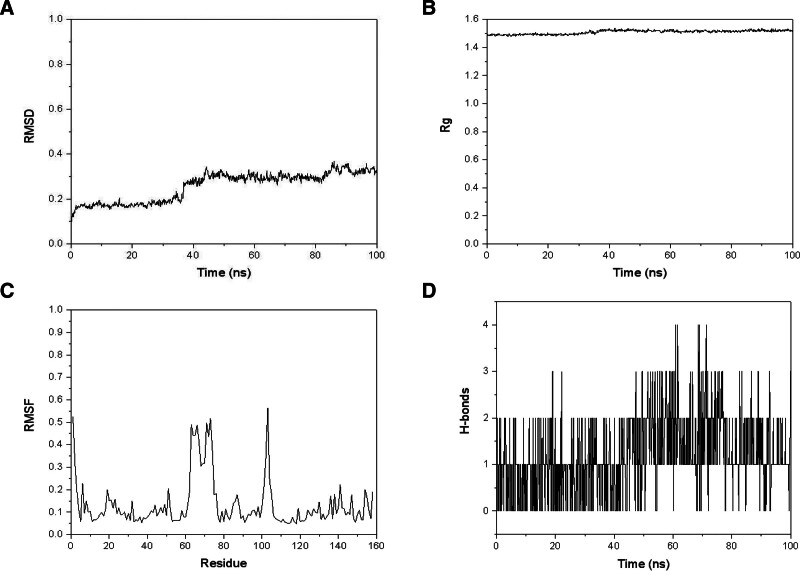
Molecular dynamics simulation of formononetin–MMP9 complex. (A) RMSD of formononetin–MMP9 complex, (B) Rg of formononetin–MMP9 complex, (C) RMSF of formononetin–MMP9 complex, (D) hydrogen bonds between formononetin and MMP9. MMP9 = matrix metalloproteinase-9, Rg = radius of gyration, RMSD = root mean square deviation, RMSF = root mean square function.

To further confirm the stability of the protein-ligand binding, an MD simulation of formononetin-MMP9 was performed. As shown in Figure 11A, in the 100 ns simulation, the RMSD value of the complex fluctuated slightly in the first 10 ns of the simulation. However, as the simulation time extended, the fluctuation amplitude of the RMSD value became smaller and gradually became stable. The final RMSD value was 0.3233 nm. As shown in Figure 11B, the Rg value of the composite system was between 1.48 and 1.54 nm, with a small fluctuation range. At 100 ns, the Rg value of the complex was 1.50871 nm. Combining the RMSD value and Rg value, the system quickly reached equilibrium in the 100 ns simulation, and the system tended to be stable and converged.

RMSF reflects the flexibility of amino acid positions. As shown in Figure 11C, the RMSF values of the complexes are all <0.6 nm, and the overall fluctuation was small. The fluctuations of Num60 to 80 and 100 to 110 were significantly larger than those of other residues.

In addition, we analyzed the hydrogen bond formed between MMP9 and formononetin. As shown in Figure 11D, in the 100 ns molecular dynamics simulation, the ligand and the protein formed hydrogen bond interactions. The numbers of hydrogen bonds formed between MMP9 and formononetin were between 0 to 4. In 100 ns, the average number of hydrogen bonds was 1.1778 ± 0.8039.

We analyzed the binding free energy of formononetin–MMP9 complex. Among them, van der waals energy < 0 and electrostatic energy < 0 indicated that both hydrophobic interaction and electrostatic energy were conducive to binding. The final total free energy was ‐33.74 ± 3.68 kcal/mol, as shown in Table 7.

**Table 7:** The binding energy between formononetin and MMP9.

Complex	ΔE_VDW_ (Kcal/mol)	ΔE_ELEC_ (Kcal/mol)	ΔG_GB_ (Kcal/mol)	ΔG_SURF_ (Kcal/mol)	ΔG_GAS_ (Kcal/mol)	ΔG_SOLV_ (Kcal/mol)	ΔG_Bind_ (Kcal/mol)
Formononetin-MMP9	‐41.63 ± 3.44	‐15.94 ± 3.76	28.43± 2.96	‐4.6± 0.24	‐57.57 ± 4.09	23.82± 2.97	‐33.74 ± 3.68

ΔE_ELEC_ = electrostatic energy, ΔE_VDW_ = Van der waals energy, ΔG_Bind_ = total free energy, ΔG_GAS_ = total gas phase free energy, ΔG_GB_ = polar solvation energy, ΔG_SOLV_ = total solvation free energy, ΔG_SURF_ = nonpolar solvation energy, MMP9 = matrix metalloproteinase-9.

## 4. Discussion

CVA belongs to “cough” in traditional Chinese medicine (TCM). According to TCM, the accumulation of wind and phlegm in the lungs and the downregulation of lung and spleen function, particularly the descending and clearance functions of the lungs, are the fundamental pathogenesis of CVA. CM treatment can eliminate wind and phlegm, strengthen the function of spleen, warm the lung, and relieve cough and asthma. The results of data mining indicated that ephedrae herba (Mahuang), Armeniacae semen amarum (Kuxingren), Glycyrrhizae radix et rhizoma (Gancao) were the most frequently herbs used in the treatment of CVA in children. Association rule analysis revealed that “Glycyrrhizae Radix Et Rhizoma (Gancao)–Ephedrae Herba (Mahuang)–Armeniacae Semen Amarum (Kuxingren)” with highest support and confidence was the core herb combination. These 3 herbs comprise the San’ao Decoction in “Taiping Huimin and Prescription Bureau.” Ephedrae Herba (Mahuang) belongs to the lung and bladder meridians, which is a mild herb characterized by a pungent, bitter taste. “Bencao Zhengyi” pointed out: “Ephedrae Herba (Mahuang) has light clearing and floating upward properties and it is the best herb for treating exogenous diseases. It is special for releasing lung qi and alleviating lung depression. Although it is said to relieve the surface, it is actually to open the lung. Although it is said to dissipate cold, it is actually to relieve evil, and the wind cold solidifies outside.” In other words, ephedrae Herba (Mahuang) eliminates the fur of the closed, down regulate the inverse lung qi, compounding the lung loss. Armeniacae semen amarum (Kuxingren) has a bitter, warm taste, and it is associated with the lung and large intestine meridian. Just as the “Bencao Xinbian” says: “It can relieve shortness of breath in chest and relieve cough.” Glycyrrhizae radix et rhizome (Gancao) has a sweet and flat flavor, which can direct reach the heart, lung, spleen, and stomach meridians. It has many functions such as tonifying middle qi, eliminating phlegm and relieving cough, easing drug properties, and so on. The combination of these 3 herbs can alleviate wind, external symptoms, and cough, which is consistent with the treatment principle for CVA in children. Clinical study have found that San’ao decoction can significantly alleviate the clinical symptoms of CVA in children, and the total effective rate is significantly higher than that of control group.^[11,12]^

The pathophysiology of CVA in children is similar to conventional asthma, which is strongly associated to airway inflammation, airway hyperresponsiveness, and airway remodeling.^[13]^ Based on the results of network pharmacology, we found that the major active ingredients of the core herb combination were quercetin, kaempferol, luteolin, formononetin, beta-sitosterol, isorhamnetin, naringenin, etc. Quercetin possesses numerous therapeutic properties, including antioxidant, antiviral, and anti-inflammatory effects. Relevant literature showed that quercetin can significantly decrease the expression levels of IL-4, IL-5, IL-6, IgE in peripheral blood and NLRP3, ASC, and caspase-1 proteins in lung tissue of rats, reducing airway inflammation and alleviating asthma symptoms.^[14–17]^ Epithelial-to-mesenchymal plays a vital role in the progression of airway remodeling. Kaempferol suppresses fibrotic airway remodeling induced by endotoxin or allergen via blocking TGF-β and PAR-1 signal pathways.^[18]^ Luteolin has anti-inflammatory, antiallergic, and immune enhancing effects. Experimental studies have found that luteolin can alleviate airway hyperresponsiveness by reducing of eosinophils, neutrophils, lymphocytes, and other inflammatory cells in the alveolar lavage fluid of asthma model mice.^[19,20]^ Formononetin and beta-sitosterol have been widely used in the treatment of allergic and inflammatory diseases.^[21–23]^ Formononetin can reduce inflammation of the airways and relieve lung oxidative damage through inhibiting leukocyte infiltration, overexpression of multiple cytokines, and collagen deposition in bronchial and lung tissues of asthmatic mice.^[24]^ Beta-sitosterol can mitigate the oxidative injury to lung tissue of rats by regulating the Th17/Treg ratio in rats with allergic asthma.^[25]^ Isorhamnetin may reduce the inflammation, proliferation, and migration of bronchial epithelial cell induced by endotoxin, exerting an anti-asthma effect.^[26]^ Naringenin is a citrus flavonoid with various biological activities, which plays an anti-allergic asthma effect through anti-inflammatory, antioxidant, and improving airway remodeling.^[27,28]^ Taken together, the main active ingredients in the core medication combination have good effect on children with CVA by reducing inflammation, allergies, regulating the immune system, and ameliorating airway remodeling.

PPI network analysis manifested that AKT1, TNF, IL6, VEGFA, JUN, IL1B, MMP9, etc were the primary targets of the core herb combination in the treatment of CVA in children. AKT1 is a serine/threonine protein kinase implicated in a variety of BPs, including cell metabolism, survival, proliferation, growth, insulin signal transduction, and angiogenesis. Clinical studies have indicated that the gene expression of AKT1in peripheral blood mononuclear cells in children with bronchial asthma is significantly increased during the attack and remission stages.^[29,30]^ Animal experiments found that the level of transcription of AKT1 in lung tissue of rats with bronchial asthma was increased, and budesonide inhalation treatment could significantly improve clinical symptoms via decreasing the level of transcription of AKT1.^[31]^ TNF, IL6, and IL1B, as important inflammatory factors, play an important role in the pathogenesis of CVA in children.^[32]^ The inflammation of airway can be alleviated by inhibiting the expression of TNF, IL6, and IL1B in the lung tissue of mice with CVA.^[33]^ As a key regulatory factor in promoting airway vascular growth, inhibiting the expression of VEGFA in lung tissue of asthmatic mice can significantly relieve airway inflammation and collagen deposition.^[34]^ JUN is involved in multiple BPs such as cell proliferation, differentiation and apoptosis. Related researches have shown that JUN subunit may be related to chronic airway inflammation in asthmatic rats, and it may be a novel target for the treatment of asthma.^[35]^ MMP9 is a member of the protease family that specifically degrades extracellular matrix, which plays an important role in airway remodeling.^[36]^ According to several articles, decreasing the expression of MMP9 in lung tissues of asthmatic rats can effectively inhibit airway remodeling and relieve clinical symptoms.^[37]^ In summary, we speculated that the core herb combination may exert therapeutic effect in treating CVA in children by inhibiting airway inflammation and hyperresponsiveness, as well as improving airway remodeling.

GO enrichment analysis showed that the core herb combination might play a therapeutic effect by regulating response to lipopolysaccharide, response to oxidative stress, response to hypoxia, and response to metal ion and other BPs. KEGG enrichment analysis suggested that TNF signaling pathway, IL-17 signaling pathway, PI3K-Akt signaling pathway, apoptosis, human cytomegalovirus infection, and Epstein-Barr virus infection were the main pathways for the core herb combination to treat CVA in children. IL-17 signaling pathway contributes to the occurrence and development of inflammatory immune diseases.^[38]^ The level of transcription of IL-17 in serum and airway of asthma patients is markedly elevated.^[39]^ TNF signaling pathway can regulate cell death and inflammatory response, which is closely related to the pathogenesis of allergic asthma.^[40]^ PI3K-Akt signaling pathway is involved in the regulation of cell growth, differentiation, survival and apoptosis. PI3K-Akt signaling pathway and apoptosis signaling pathway play a pivotal role in various pathological stages of asthma airway inflammation, airway hyperreactivity, and airway remodeling.^[41–43]^ Human cytomegalovirus infection signal pathways and Epstein-Barr virus infection signal pathways have been demonstrated that they play a key role in immunological modulation.^[44,45]^

The results of molecular docking indicated that the active components could stably bind to the target of core protein. The docking scores of formononetin, luteolin, naringenin, and quercetin with MMP9 were all <‐10 kcal/mol and they formed strong hydrogen bonds and hydrophobic interactions. The ADMET analysis showed that formononetin, luteolin, naringenin, and quercetin had high gastrointestinal absorption, no AMES toxicity, hepatotoxicity, and skin sensitization. In the MD simulation, the formononetin-MMP9 complex was relatively stable and hydrogen bond interactions could be formed.

There were still some limitations in this article. Firstly, in the data mining section, only prospective clinical studies were included, and the total effective rate was ≥80%. Although the reliability of the data was ensured, some literatures and prescriptions were excluded, arising possible deviations in the results. Secondly, the targets of active ingredients of herbs and disease mainly came from databases, and continuous updates in databases might lead to inaccurate results in this study. Thirdly, the research findings regarding mechanism of CM for children with CVA were derived from network pharmacology, molecular docking, and MD simulation. It still required experimental confirmation.

## 5. Conclusion

In summary, this article explored medication rules and molecular mechanism of CM against CVA in children using data mining, network pharmacology, molecular docking, and MD simulation. It was discovered during data mining that “Glycyrrhizae Radix Et Rhizoma (Gancao)–Ephedrae Herba (Mahuang)–Armeniacae Semen Amarum (Kuxingren)” was the core herb combination with highest support and confidence. Network pharmacology found that quercetin, kaempferol, luteolin, formonetin, beta-sitosterol, isorhamnetin, naringenin, and other components in the core herb combination might inhibit the airway inflammation of CVA in children by regulating AKT1, TNF, IL6, VEGFA, JUN, IL1B, MMP9, and other core targets. Multiple signal pathways such as IL-17 signaling pathway, TNF signaling pathway, PI3K-Akt signaling pathway were associated with airway hyperresponsiveness, airway remodeling and other pathological stages, reflecting the advantages of multi-component, multi-target, and multi-level collaborative treatment of TCM. The results were verified by molecular docking and MD simulation.

## Author contributions

**Conceptualization:** Yuan Ma, Fengping Sun.

**Data curation:** Yuan Ma.

**Funding acquisition:** Fengping Sun.

**Investigation:** Yingjie Hu, Jing Li.

**Methodology:** Yuan Ma, Yue Ding.

**Supervision:** Yingjie Hu, Liyang Duan.

**Validation:** Yuan Ma, Fengping Sun.

**Writing – original draft:** Yuan Ma.

**Writing – review & editing:** Yuan Ma, Fengping Sun.
